# Image-Based Mobile System for Dietary Management in an American Cardiology Population: Pilot Randomized Controlled Trial to Assess the Efficacy of Dietary Coaching Delivered via a Smartphone App Versus Traditional Counseling

**DOI:** 10.2196/10755

**Published:** 2019-04-23

**Authors:** Brian G Choi, Tania Dhawan, Kelli Metzger, Lorraine Marshall, Awdah Akbar, Tushina Jain, Heather A Young, Richard J Katz

**Affiliations:** 1 Department of Medicine School of Medicine & Health Sciences The George Washington University Washington, DC United States; 2 Department of Epidemiology and Biostatistics Milken Institute School of Public Health The George Washington University Washington, DC United States

**Keywords:** Mediterranean diet, telemedicine, cardiovascular disease, randomized controlled trial

## Abstract

**Background:**

Randomized controlled trials conducted in Mediterranean countries have shown that the Mediterranean diet lowers adverse cardiovascular events. In the American population, diet remains the biggest uncontrolled risk factor for cardiovascular disease.

**Objective:**

This study aimed to test the hypothesis that asynchronous dietary counseling supplied through a custom smartphone app results in better adherence to a Mediterranean diet in a non-Mediterranean population than traditional standard-of-care (SOC) counseling.

**Methods:**

In total, 100 patients presenting to the cardiology clinic of an academic medical center were randomized to either the SOC or smartphone app-based experimental (EXP) Mediterranean diet intervention after informed consent and 1 hour of individual face-to-face dietary counseling with a registered dietitian. Participants in EXP received a custom smartphone app that reinforced the Mediterranean diet, whereas participants in SOC received 2 additional sessions of in-person dietary counseling with the registered dietitian—30 min at 1 month and 30 min at 3 months. Preexisting knowledge of a Mediterranean diet was measured by the validated Mediterranean Diet Score (MDS) instrument. Baseline height, weight, blood pressure (BP), and laboratory biomarkers were collected. At 1, 3, and 6 months, participants presented for a follow-up appointment to assess compliance to the Mediterranean diet using the MDS as well as a patient satisfaction survey, BP, and weight. Repeat laboratory biomarkers were performed at 3 and 6 months.

**Results:**

Enrolled participants had a mean age with SE of 56.6 (SD 1.7) for SOC and 57.2 (SD 1.8) for EXP; 65.3% of SOC and 56.9% of EXP were male, and 20.4% of SOC and 35.3% of EXP had coronary artery disease. There were no significant differences between EXP and SOC with regard to BP, lipid parameters, hemoglobin A_1c_, or C-reactive protein (CRP). Participants in EXP achieved a significantly greater weight loss on average of 3.3 pounds versus 3.1 pounds for participants in SOC, *P*=.04. Adherence to the Mediterranean diet increased significantly over time for both groups (*P<*.001), but there was no significant difference between groups (*P*=.69). Similarly, there was no significant difference in diet satisfaction between EXP and SOC, although diet satisfaction increased significantly over time for both groups. The proportion of participants with high Mediterranean diet compliance (defined as the MDS ≥9) increased significantly over time *(P<*.001)—from 18.4% to 57.1% for SOC and 27.5% to 64.7% for EXP; however, there was no significant difference between the groups.

**Conclusions:**

Both traditional SOC counseling and smartphone-based counseling were effective in getting participants to adhere to a Mediterranean diet, and these dietary changes persisted even after counseling had ended. However, neither method was more effective than the other. This pilot study demonstrates that patients can change to and maintain a Mediterranean diet with either traditional or smartphone app-based nutrition counseling.

**Trial Registration:**

ClinicalTrials.gov NCT03897426;https://clinicaltrials.gov/ct2/show/NCT03897426

## Introduction

Cardiovascular disease (CVD) has been the leading cause of death every year since 1918 in the United States and currently accounts for 1 in every 3 deaths [1]. Diet is the most uncontrolled risk factor for cardiovascular health among US adults; per the 2015-2020 Dietary Guidelines for Americans, approximately 75% of the population follows an unhealthy eating pattern and less than 2% of Americans have an ideal diet [2]. These suboptimal dietary habits are the leading cause of mortality and disability-adjusted life years lost, greater than smoking, obesity, physical inactivity, high cholesterol, hypertension, or diabetes [3].

Randomized controlled trials (RCTs) have shown superiority of the Mediterranean diet in both high-risk primary prevention and secondary prevention populations at risk for CVD [4]. The Mediterranean diet is typified by olive oil, nuts, vegetables, legumes, fish, white meat, and wine, and the Prevención con Dieta Mediterránea (PREDIMED) study showed a 30% reduction in major adverse cardiovascular events for patients at high risk of developing atherosclerotic disease when they received a Mediterranean diet enriched with olive oil and nut consumption [5]. The Lyon Diet Heart Study enrolled patients after a first myocardial infarction, and those randomized to receiving a Mediterranean diet had improved survival and fewer myocardial infarctions than those on a usual prudent diet [6]. These studies, however, were conducted in Mediterranean countries and whether this diet can be achieved in non-Mediterranean populations with similar benefits remains to be elucidated [7]. If a Mediterranean diet can be successfully implemented and maintained in a high-risk American population, substantial reductions in cardiovascular morbidity and mortality may follow [8].

Our study tested the hypothesis that experimental asynchronous dietary counseling provided through a custom smartphone app (EXP) would improve implementation and compliance to a Mediterranean diet and secondarily evaluated the effect of this intervention on markers of cardiovascular risk and patient satisfaction compared with standard-of-care (SOC) counseling, that is, face-to-face counseling from a registered dietitian (RD).

## Methods

### Recruitment

**Figure 1:**
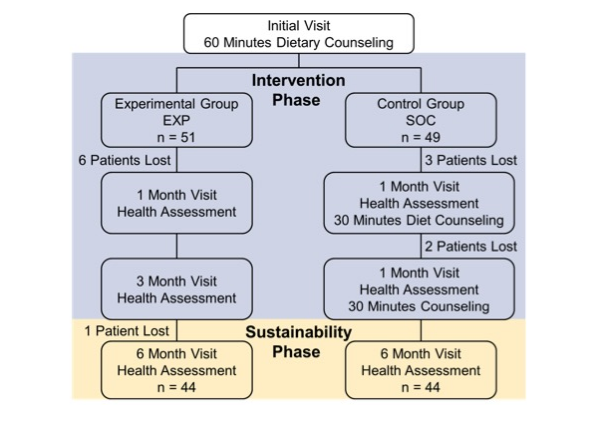
Randomized controlled trial design. Of the patients that dropped out of the study, 6 participants (4 standard-of-care, SOC and 2 experimental arm, EXP) did not show up to scheduled appointments, 1 participant (EXP) withdrew from the study because the food was “too expensive,” 3 participants (all EXP) withdrew because the app failed to work on their phone, and 2 participants (1 SOC and 1 EXP) were no longer interested in participating in the study and did not give a clarifying reason.

A total of 100 patients were recruited from the cardiology clinic of an academic medical center in Washington, DC, for this RCT (Figure 1) as cardiology patients may be the ones most likely to benefit from dietary modification to the Mediterranean diet.

All patients scheduled for the clinic were screened via query of the electronic medical record system to see if those potential study participants would meet the eligibility criteria, recruitment posters were posted in exam rooms of the clinic, and those candidates meeting the eligibility criteria were further screened by their cardiologist to see if they would qualify. Potential candidates were approached for participation by a research team member at the time of their clinic visit in coordination with the participant’s cardiologist. The inclusion criteria included ongoing cardiology care with a cardiologist that is expected to continue for at least 6 months (to limit the potential for participants in being lost in follow-up), personal ownership of an Android- or iOS (iPhone operating system)-based smartphone with a data plan, English language proficiency, demonstration of the ability to download and install the app, aged at least 18 years, and a minimum fifth grade literacy level per the Rapid Estimate of Adult Literacy in Medicine [9]. The exclusion criteria were clinical instability at the time of enrollment; comorbid medical disease that would preclude the ability to participate in a nutrition intervention study (eg, digestive disease with fat intolerance); life expectancy less than 5 years; severe neurologic, psychiatric, or endocrine abnormalities; immunodeficiency or HIV-positive status; illegal drug use; alcoholism or daily alcohol intake >80 grams/day (ie, 5 12-ounce glasses of beer, 5 5-ounce glasses of wine, or 5 1.5-ounce glasses of spirits); body mass index (BMI) >40 kg/m^2^ (as these patients should be on a weight-reducing diet instead); inability or unwillingness to change dietary habits per patient report; inability or unwillingness to adhere to a Mediterranean diet (eg, religious or moral reasons); disorders of chewing or swallowing; allergies to major components of the Mediterranean diet (eg, olive oil and nuts); participation in any drug trial or use of any investigational drug within the past month; institutionalized or nonambulatory status; lack of autonomy; no stable address; acute infection or inflammation (patients could be reconsidered when/if the condition resolves); and patients currently pregnant, breastfeeding, or planning to become pregnant.

Those patients who met the enrollment criteria were invited to participate by a study coordinator. Patients who wished to enroll signed the informed consent and then received 1 hour of face-to-face counseling on the Mediterranean diet from an RD. During the initial visit, the RD reviewed the rationale of the Mediterranean diet, detailed the expected diet and lifestyle modifications involved, and developed a contract for negotiated change. Participants were provided with shopping lists, weekly plans, and recipes, and the RD administered a quantitative questionnaire of Mediterranean diet compliance, the Mediterranean Diet Score (MDS). Initial blood pressure (BP), weight, total cholesterol, high-density lipoprotein (HDL) cholesterol, low-density lipoprotein (LDL) cholesterol, triglycerides, hemoglobin A_1c_ (HbA_1c_), and high-sensitivity C-reactive protein (CRP) were obtained. At the end of the visit, the participant was randomized to either the app-based intervention arm (EXP) or SOC based upon a predetermined random sequence. Participants randomized to the intervention arm were given an instruction manual, directed to a website for additional information on how to use the app, and had the smartphone app set up on their mobile device to establish connectivity with the RD. As reimbursement, patients in both treatment groups received a small stipend to cover the cost of travel for each visit as well as a nominal gift card to a local grocery store that carried foods included in the Mediterranean diet.

### Standard-of-Care Arm

Participants could initiate contact and receive brief telephone-based counseling with their assigned RD at any time during the study; however, the RD would not initiate additional face-to-face dietary counseling until the 1-month and 3-month visit. At 1 month, participants returned for a 30-min follow-up counseling session with the RD during which dietary recall was reviewed, education reinforced, and strategies for improvement developed. Repeat BP and weight measurements were obtained. The MDS was readministered, interval medical history events were reviewed, and satisfaction with SOC and the Mediterranean diet was assessed.

At 3 months, the RD administered a final 30 min of dietary counseling; dietary recall was again reviewed, education reinforced, and strategies for improvement further developed. Repeat BP, weight, fasting total cholesterol, HDL cholesterol, LDL cholesterol, triglycerides, HbA_1c_, and high-sensitivity CRP were also obtained during the 3-month visit. The MDS was readministered, interval medical history events were again reviewed, and satisfaction with SOC and the Mediterranean diet was reassessed.

The participant then entered a sustainability phase during which the RD could not initiate contact. At 6 months, the participant had a final visit with a research coordinator. Repeat BP, weight, fasting total cholesterol, HDL cholesterol, LDL cholesterol, triglycerides, HbA_1c_, and high-sensitivity CRP were obtained. The MDS was readministered. Interval medical history events were reviewed. Satisfaction with SOC and the Mediterranean diet was assessed. Participants were also asked if they received any additional dietary counseling not supported through the study.

### Intervention Arm

**Figure 2:**
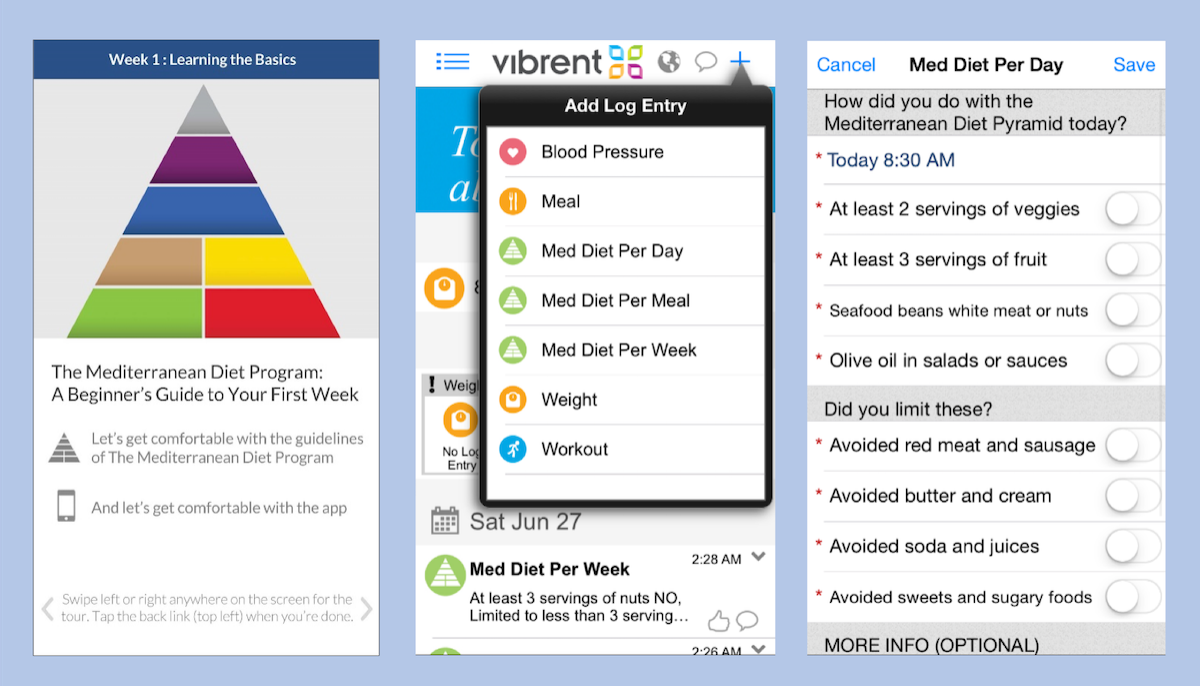
App-based diet education and tracking to encourage Mediterranean diet compliance. From left to right, the first image illustrates the app-based learning material on the Mediterranean diet, the second image shows a drop-down menu of self-assessment tools offered by the app, and the third displays one of the self-assessment tools—a Mediterranean diet log to record daily compliance.

Participants in the intervention arm (EXP) were allotted 60 min with the RD through the customized smartphone app (Figure 2) and encouraged to fully utilize these 60 min in the 3 months following enrollment. The app was created by Vibrent Health (Fairfax). Vibrent Health consulted with the research team on the app’s design, and Vibrent Health was free to modify the app throughout the course of the study to load new educational content provided by the research team, refresh challenges, and maintain compatibility with mobile phone operating systems and new smartphone designs. Only time spent by the RD interacting with the participant through the app counted against the allotted time. At the initial visit, the participants received the same educational handouts provided to the SOC arm, but also had this content preloaded onto the app. The app included weekly challenges to encourage dietary modification, and either the RD or the patient could initiate contact through the app. Participants were encouraged to use the app to take pictures of their food, document meals and amounts consumed, ask questions to the RD, document exercise, and monitor their BP and track it in the log if another provider recommended that it be recorded. The challenges were for patients to challenge themselves (ie, they did not compete against other participants). Examples of challenges included increasing daily servings of vegetables or exercising daily. Additional face-to-face counseling was not offered by the RD. The allotted time that was not used in the first 3 months could be used after 3 months, but no counseling was provided after 6 months. If the participants used all of their allotted time, they could continue to initiate contact with their RD through traditional means (eg, by telephone).

**Figure 3:**
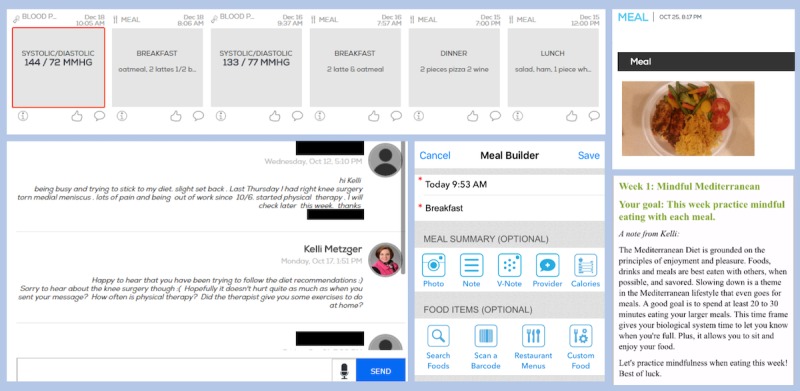
Asynchronous counseling and meal-logging tools of the customized smartphone app. From left to right, in the top row, the first image shows the app’s timeline view of various logs (eg, blood pressure and meals) and the second image represents the app’s photo log feature via the meal a patient photographed; in the bottom row, the first image illustrates the registered dietitian (RD)-patient interface for app-based nutritional counseling, the second depicts the app’s Meal Builder feature with its meal- and food-logging options, and the third exemplifies the app’s weekly challenge feature via a sample counseling note sent by the RD through the app.

Participants in EXP received dietary counseling from their RD via the app in the context of their meal logs (Figure 3). The amount of time the RD spent responding to a participant through the provider portal was tracked to ensure it fell within the 60 min limit. Participants received the RD’s feedback directly to their mobile phone to review at their own convenience. After the 6-month visit, the app no longer had RD access and support was not continued.

Similar to the SOC group, participants in EXP had a follow-up visit with a research coordinator at 1 month, 3 months, and 6 months following enrollment. At each visit, interval medical history events were reviewed, the MDS was readministered, and repeat BP and weight obtained. Participant satisfaction with the intervention and Mediterranean diet was also assessed at each visit. Fasting total cholesterol, HDL cholesterol, LDL cholesterol, triglycerides, HbA_1c_, and high-sensitivity CRP were measured at the 3-month and 6-month visits.

The MDS, the 14-point quantitative score of adherence to the Mediterranean diet, was the primary endpoint of this RCT. Secondary endpoints included BP, weight, fasting lipid parameters (total cholesterol, HDL cholesterol, LDL cholesterol, and triglycerides), HbA_1c_, high-sensitivity CRP, participant satisfaction with the intervention versus usual care, and percent of participants achieving high compliance with a Mediterranean diet (defined as MDS ≥9).

### Statistical Analysis

The sample size was determined using the study’s hypotheses and primary endpoint of the MDS. Given an estimated baseline MDS of 4.5 with an SD of 2 units, a total sample size of 100 (n=50 per group) would have adequate power (93%) to detect a difference of 1.4 units in the MDS between the SOC and intervention arm. Even after an assumed attrition rate of 20% overall (total n=80), the study would retain a power of at least 80% to detect a minimum difference of 1.4 units between SOC and EXP.

Descriptive statistics of baseline characteristics plus primary and secondary endpoints at baseline and certain time points were calculated for both groups, and the 2 groups were compared using the chi-square test, Fisher exact test, Wilcoxon signed rank, or 2-sample test, as appropriate. The descriptive statistics for app usage data were also summarized for EXP participants who used each portion of the app.

Repeated measures analysis of variance was used to compare primary and secondary outcomes over time and across groups. Wilcoxon signed rank test was used to assess differences in the participant satisfaction score (PSS) between SOC and EXP at individual time points. As a secondary endpoint, the study also compared the proportion of participants that achieved a high compliance (MDS ≥9) with the Mediterranean diet between the 2 groups; McNemar’s chi-square test was used to determine if there were differences between the 2 groups from baseline to last visit.

Differences in MDS from baseline to last visit were calculated and compared between SOC and EXP within subgroups of sex, diabetes, alcohol use, smoking, and atherosclerotic cardiovascular disease (ASCVD) using analysis of variances. App usage data were broken into quartiles based on total app usage time (0 to 8.5 min; 8.5 to 16 min; 16 to 30 min; or more than 30 min); high (>16 min) and low (≤16 min) groups for RD usage; and dichotomous (yes/no) groups for challenge, weight log, workout log, and BP log usage. Repeated measures analysis of variance was used to examine differences in the MDS across app usage groups and over time. Chi-square was employed in examining differences in proportions of weight loss, HDL increase, and lowered systolic BP across app usage groups.

Dropouts were analyzed using intention-to-treat methodology so they would be analyzed in the group they started in up to the period at which they dropped out. Differences in percentages of dropouts were also examined to determine if outcomes differed across groups.

The study was reviewed and approved by the institutional review board. At the time this study was started, trial registration was not required at Clinicaltrials.gov but has since been registered by the study sponsor Vibrent Health (Unique Protocol ID HHSN261201400053C_SBIR 308).

## Results

The mean age was 56.6 (SE 1.7) years for SOC and 57.2 (SE 1.8) years for EXP. There was a significantly higher number of participants with diabetes at baseline in SOC (*P*=.01). For the other baseline characteristics, there was no significant difference between SOC and EXP (Tables 1 and 2). There were 12 dropouts over the course of the study (Figure 1).

**Table 1:** Baseline characteristics of the study population.

Variable		Standard-of-care arm, n (%)	Experimental arm, n (%)
**Gender**			
	Female	17 (34.7)	22 (43.1)
	Male	32 (65.3)	29 (56.9)
**Medical history**			
	Diabetes^a^	10 (20.4)	2 (3.9)
	Atherosclerotic CVD^b^	10 (20.4)	18 (35.3)
	Previous myocardial infarction	8 (16.3)	10 (19.6)
	Previous revascularization	12 (24.5)	18 (35.3)
	Previous stroke or transient ischemic attack	2 (4.1)	3 (5.9)
	Peripheral vascular disease	1 (2.0)	1 (2.0)
	Other CVD	39 (92.9)	38 (90.5)
**Alcohol use**			
	Previous	1 (2.0)	2 (4.1)
	Current	32 (68.1)	39 (79.6)
**Tobacco use**			
	Never	29 (59.2)	32 (62.8)
	Previous	13 (26.5)	14 (27.5)
	Current	4 (8.2)	2 (4.0)

^a^*P*=.01.

^b^CVD: cardiovascular disease.

**Table 2:** Mean and SE for outcome variables by treatment group and time of visit.

Group		Initial, mean (SE)	1 month, mean (SE)	3 month, mean (SE)	6 month, mean (SE)	*P*_overtime_	*P*_group_
**Systolic BP^a^** **(mmHg)**							
	SOC^b^	128.1 (2.9)	125.4 (2.4)	130.3 (2.9)	128.7 (2.5)	.43	.34
	EXP^c^	129.6 (2.3)	128.5 (2.2)	128.2 (2.2)	129.5 (2.5)	.43	.34
**Diastolic BP (mmHg)**							
	SOC	78.2 (1.1)	77.8 (1.3)	79.5 (1.4)	79.2 (1.5)	.75	.27
	EXP	78.3 (1.1)	79.2 (1.5)	77.8 (1.5)	78.7 (1.5)	.75	.27
**Body mass index (kg/m^**2**^** **)**							
	SOC	30.8 (0.6)	30.4 (0.7)	30.7 (0.7)	30.5 (0.7)	.02	.03
	EXP	29.5 (0.6)	29.0 (0.6)	28.9 (0.6)	28.7 (0.7)	.02	.03
**Total cholesterol (mg/dL)**							
	SOC	171.9 (5.4)	—^d^	167.1 (5.1)	172.0 (6.4)	.19	.90
	EXP	161.6 (5.4)	—	157.3 (5.8)	159.3 (6.1)	.19	.90
**Triglyceride (mg/dL)**							
	SOC	104.2 (8.0)	—	98.0 (6.5)	97.5 (7.3)	.29	.71
	EXP	107.3 (9.2)	—	102.8 (8.4)	99.8 (8.5)	.29	.71
**High-density lipoprotein (mg/dL)**							
	SOC	54.1 (2.4)	—	54.9 (2.7)	54.3 (2.5)	.40	.12
	EXP	53.8 (2.4	—	52.9 (2.3)	55.3 (2.8)	.40	.12
**Very low-density lipoprotein (mg/dL)**							
	SOC	22.9 (2.7)	—	19.5 (1.3)	19.5 (1.5)	.31	.71
	EXP	21.4 (1.8)	—	20.5 (1.7)	19.9 (1.7)	.31	.71
**Low-density lipoprotein (mg/dL)**							
	SOC	94.5 (5.3)	—	92.7 (4.7)	98.3 (5.4)	.35	.58
	EXP	86.4 (4.2)	—	83.9 (4.6)	84.1 (4.9)	.35	.58
**Hemoglobin A_1c_ (%)**							
	SOC	6.2 (0.2)	—	6.0 (0.1)	6.1 (0.1)	.94	.45
	EXP	5.8 (0.1)	—	5.9 (0.1)	5.8 (0.1)	.94	.45
**C-reactive protein (mg/dL)**							
	SOC	3.3 (0.9)	—	2.5 (0.4)	2.2 (0.4)	.34	.29
	EXP	1.9 (0.3)	—	1.6 (0.2)	1.5 (0.3)	.34	.29
**Diet Satisfaction Score (units)**							
	SOC	—	0.42 (0.04)	0.59 (0.05)	0.53 (0.05)	<.001	.29
	EXP	—	0.36 (0.05)	0.46 (0.05)	0.50 (0.05)	<.001	.29

^a^BP: blood pressure.

^b^SOC: standard-of-care arm.

^c^EXP: experimental arm.

^d^Not applicable.

**Figure 4:**
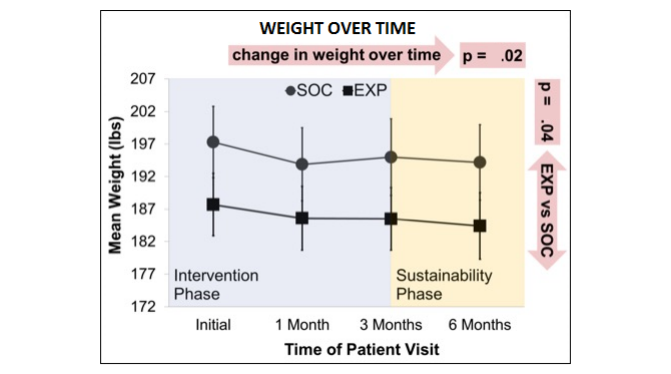
Change in weight over time (mean with SE). SOC: standard-of-care arm; EXP: experimental arm.

**Figure 5:**
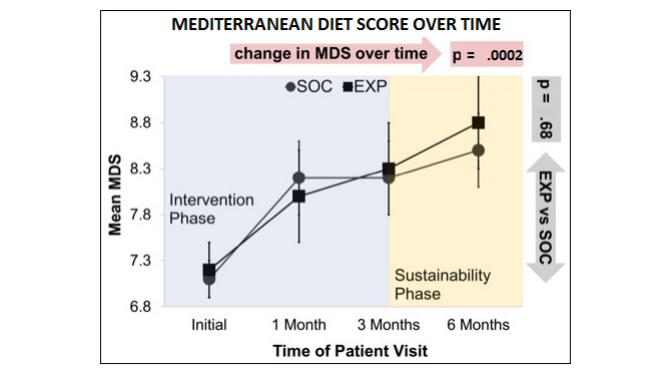
Compliance with the Mediterranean diet overtime (mean with SE). MDS: Mediterranean diet score; SOC: standard-of-care arm; EXP: experimental arm.

From baseline to final visit, there was a significant decrease in BMI (Table 2) and weight (Figure 4) that varied by group—participants in EXP experienced a slightly larger decrease in weight of 3.3 pounds versus 3.1 pounds (*P*=.04) and BMI of 0.8 versus 0.3 (*P*=.03). In addition, the MDS (Figure 5) and Diet Satisfaction Score (Table 2) increased significantly over time for both SOC and EXP, although there was no statistically significant difference in these trends between the 2 groups. Changes in the other outcome variables analyzed were not statistically significant over time or between the 2 treatment groups (Table 2).

**Figure 6:**
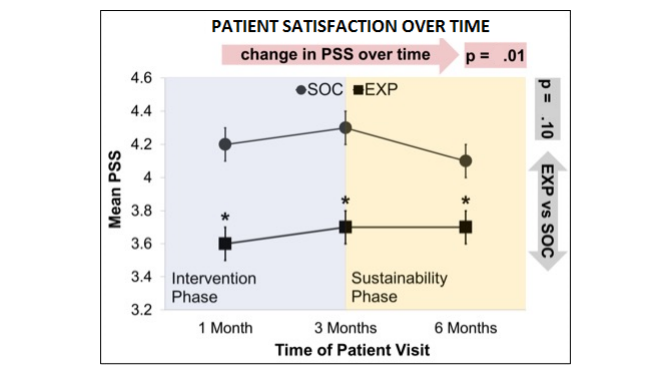
Patient satisfaction over time (mean with SE). PSS: patient satisfaction score; SOC: standard-of-care arm; EXP: experimental arm.

There was a significant change in average PSS over time (Figure 6), but the change over time did not vary by group. However, at each time point, SOC had a significantly higher average patient satisfaction score compared with EXP (*P*=.01).

**Figure 7:**
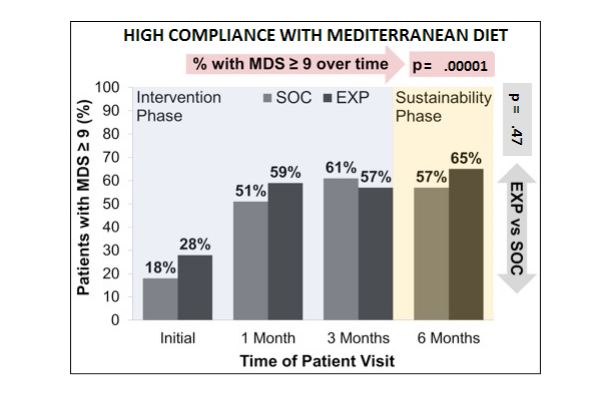
Proportion of patients achieving high compliance with the Mediterranean diet over time. MDS: Mediterranean diet score; SOC: standard-of-care arm; EXP: experimental arm.

For both SOC and EXP, the proportion of participants that achieved high compliance (defined as MDS ≥9) with the Mediterranean diet increased significantly (McNemar’s chi-square *P<*.0001) overtime from the initial visit to the 6-month visit (Figure 7). However, when comparing SOC with EXP at each time point, there were no significant differences in MDS ≥9 at any of the time points (initial: *P*=.33; 1 month: *P*=.50; 3 months: *P*=.44; and 6 months: *P*=.47). There was also no significant difference in any individual component of the MDS with time (at baseline or final visit) or between the 2 groups.

There was a difference in the MDS by ASCVD (*P*=.03). In both the SOC and experimental group, participants without ASCVD showed a larger increase in the MDS over the course of the study than participants with ASCVD (*P*=.02); however, this difference was not statistically significant between the SOC and experimental group. There were no differences in change in MDS from baseline to final visit by sex, diabetes, alcohol use, or smoking.

There were no significant differences in MDS over time by app usage quartiles, high versus low RD use, or challenge participation. There was no significant difference in weight loss between participants who used the weight log versus those who did not. However, of the 14 participants who used the workout log, 12 (86%) experienced weight loss from baseline to final visit. This was significantly higher (*P*=.04) than the 20 of 39 participants (54%) not using the workout log who experienced weight loss.

Of the 15 participants who used the BP log, 9 (60%) lowered their systolic BP from baseline. This was not significantly different from the 13 of 36 participants (38%) not using the BP log who lowered their systolic BP from baseline.

## Discussion

### Principal Findings

Both traditional, SOC face-to-face counseling, and app-based dietary counseling were effective at getting participants to adhere to a Mediterranean diet in this US-based urban study of patients from a cardiology practice, and these dietary changes persisted even after counseling had ended. However, neither method of dietary counseling was more effective than the other at meeting the primary endpoint of Mediterranean diet compliance. These results were consistent whether the app was used heavily or lightly by participants in the experimental arm based on the subgroup analyses.

At each time point, participants were more satisfied with traditional counseling than with the app, but participant satisfaction regressed during the maintenance phase after traditional counseling ended. With the app, satisfaction did not regress. We speculate that this effect may be because participants were permitted to continue to use the app during the maintenance phase. Both groups showed increased satisfaction with their changed diet overall, but there were no differences in the level of satisfaction between the 2 groups.

Although the evidence suggests that participants with ASCVD derive more benefit from a Mediterranean diet based on comparison between the Lyon Diet Heart Study [4] and the PREDIMED investigation [5], participants in this study were more likely to adapt a Mediterranean diet if they did not have ASCVD. This result suggests that dietary patterns in this urban US population may be more modifiable for primary prevention purposes rather than for secondary prevention.

### Comparison With Previous Work

Asynchronous mobile health–based dietary counseling has been shown to improve dietary targets. For example, Rossi et al demonstrated that mobile phone software that included an interactive diary for diet management and enabled users to communicate with a dietitian via short text messages was successful in helping users better identify and achieve Mediterranean diet targets such as the increased consumption of fresh fruits and vegetables [10]. However, this was a single-arm longitudinal investigation that did not use a comparison with usual care and was not studied in a non-Mediterranean population. Papadaki and Scott were able to show the efficacy of a Web-based intervention in promoting key components of the Mediterranean diet in a non-Mediterranean population via a quasi-experimental study [11], but their intervention did not use a mobile health–based approach. Our research is unique in that it studies the use of a smartphone-based dietary intervention for the Mediterranean diet by way of the RCT method.

We found that the use of a mobile app for the Mediterranean diet did not result in significant differences between groups or subgroups overall, aside from weight and BMI. No other biomarkers (eg, systolic BP and HbA_1c_) indicated that the smartphone app was more successful than SOC dietary counseling at differentiating outcomes. Nevertheless, this investigation demonstrates that the Mediterranean diet can be achieved by a non-Mediterranean population. Given that dietary counseling—which was standardized in both the SOC and intervention arms—resulted in increased adherence to the Mediterranean diet for participants in both treatment groups, a mobile app–based intervention is a practical option for administering dietary counseling when delivered in conjunction with traditional dietitian-based counseling. An app-based dietary intervention may be less resource intensive for maintenance of dietary changes and still deliver the same effect as traditional counseling. In addition, this effect was not altered by app use intensity, suggesting it is the baseline functionality of the app without the RD interaction that drove this benefit. Moreover, although not designed to be a weight-reducing diet, participants who received app-based Mediterranean diet counseling lost more weight than those who received SOC dietary counseling. The subgroup analysis suggests that this weight loss effect may have been driven by the presence of an exercise/activity log within the app.

Our findings are in keeping with previous research; for example, the EVIDENT II trial also demonstrated that dietary counseling is effective in increasing adherence to the Mediterranean diet in a population of primary care patients in Spain and found that the use of a mobile app had no significant impact on this result [12]. As EVIDENT II had greater female participation, these results also support that gender did not influence the outcome, and similar to EVIDENT II, despite finding no difference in the primary endpoint, there was a signal toward increased exercise in the app group (however, in our study, there was no accelerometer data and the signal was simply reflected in exercise log usage).

### Limitations

This RCT was conducted as a pilot study. The strength of the investigation was limited by a small sample size of 100 patients. As more patients were screened than qualified for the study, biases based upon unrecorded reasons for study nonparticipation may also have influenced the outcome. In addition, the 6-month duration of the study may have been too short to demonstrate cardiovascular health benefits via parameters such as BP, lipid levels, HbA_1c_, and CRP, and this pilot study was not adequately powered to detect such an effect.

As this study was performed at a single academic medical center, the ability to extrapolate these results to community-based practices or other locations including the nonurban environment is limited. Furthermore, the typical payer mix of our institution trends toward the underserved, and the results may be different if the participant pool was enriched with those from a higher socioeconomic status.

Emphasis should be made that this study cannot determine whether any sustainable dietary modification could have been achieved in the absence of counseling from a dietitian. Indeed, the benefit appears to have been driven by the initial RD interaction which occurred in both arms of the study, emphasizing the point that apps may best be utilized as an adjunct to traditional counseling as opposed to a replacement [13].

Study design may also have impacted the strength of our results as the nature of the intervention in this RCT prevented blinding of the participants, and several findings of the study (eg, dietary compliance) relied on self-reported information. Moreover, older and elderly adults are often more resistant to behavioral change and may be less comfortable with smartphones and mobile technology than younger adults [14]; this generational and age-related bias may have distorted the measured and reported efficacy of and satisfaction with the mobile app and Mediterranean diet. As the survey instrument was administered by the dietitian in SOC at the 1- and 3-month time points, these survey responses may have been biased; however, the results at the interim time points did not differ from the final time point (when all surveys were administered by the same coordinator). Despite its limitations, this RCT is hypothesis generating and can guide strategies for improving preventative cardiology. As the effects were not different between those who used the app heavily and those who did not use the app much, it is possible that the results would have been different with a different, more engaging app given the low PSS in the experimental group.

### Conclusions

In summary, the dietary pattern of the urban US cardiology patient is modifiable to greater adherence to a Mediterranean diet, whether that dietary intervention is face-to-face or delivered via a mobile smartphone app. Supplemental features that promote weight loss within a smartphone app may, in addition, help weight loss. Although participants were less satisfied with the app, it may have presented a more time-effective means of delivering dietary counseling and resulted in less loss of effectiveness as compared with the period after completion of face-to-face counseling.

## Abbreviations

ASCVD: atherosclerotic cardiovascular disease
BMI: body mass index
BP: blood pressure
CRP: C-reactive protein
CVD: cardiovascular disease
EXP: experimental arm
HbA _1c_: hemoglobin A_1c_
HDL: high-density lipoprotein
LDL: low-density lipoprotein
MDS: Mediterranean diet score
PREDIMED: Prevención con Dieta Mediterránea
PSS: participant satisfaction score
RCT: randomized controlled trial
RD: registered dietitian
SOC: standard-of-care
